# Stage 1 Registered Report: How responsibility attributions to self and others relate to outcome ownership in group decisions.

**DOI:** 10.12688/wellcomeopenres.16480.2

**Published:** 2021-05-05

**Authors:** Matt Jaquiery, Marwa El Zein

**Affiliations:** 1 Department of Experimental Psychology, University of Oxford, Oxford, OX2 6GG, UK; 2 Institute of Cognitive Neuroscience, University College London, London, WC1N 3AZ, UK; 3 Adaptive Rationality Center, Max-Planck for Human Development, Berlin, 14195, Germany

**Keywords:** Responsibility attribution, outcome ownership, outcome valence, self-serving bias, other-serving bias, group decisions.

## Abstract

Responsibility judgements have important consequences in human society. Previous research focused on how someone's responsibility determines the outcome they deserve, for example, whether they are rewarded or punished. Here, we investigate the opposite link: How outcome ownership influences responsibility attributions in a social context. Participants in a group of three perform a majority vote decision-making task between gambles that can lead to a reward or no reward. Only one group member receives the outcome and participants evaluate their and the other players' responsibility for the obtained outcome. Two hypotheses are tested: 1) Whether outcome ownership increases responsibility attributions even when the control over an outcome is similar. 2) Whether people's tendency to attribute higher responsibility for positive vs negative outcomes will be stronger for players who received the outcome. The findings of this study may help reveal how credit attributions can be biased toward particular individuals who receive outcomes as a result of collective work.

## Introduction

How we judge people’s responsibility for the outcomes of their actions has important consequences in our society. This is true for our own responsibility as well as others’. Responsibility judgements are tightly related to whether people get rewarded or blamed for the actions they make ^1, 2^, which is crucial for the maintenance of a cooperative and fair society. In many everyday situations, whether in the workplace or with our family and friends, responsibility for outcomes is shared among several individuals because these outcomes stem from collective decisions. Collective decisions can reduce the burden of individual responsibility ^3– 5^, because people feel less responsible when performing an action as a group than when acting alone ^6– 12^.

While collective decision-making reduces overall feelings of responsibility, fluctuations in feelings of responsibility are also affected by the outcome of a decision. People tend to attribute higher responsibility to themselves for positive as compared to negative outcomes. This is known as the **self-serving bias**, where people claim more credit for positive events, while they duck responsibility for negative events ^6, 9, 11, 12^. This ‘valence bias’ however does not seem to be purely selfish: it also appears when people judge their group’s or another person’s responsibility ^13, 14^. For example, in the context of advising, people exhibit an **‘other-serving’ bias** in which they tend to credit more than blame an advisor ^13^. In line with this, people also tend to attribute more effort for higher rewards when judging someone else’s effort as compared to their own effort ^15^.

In individual contexts, the rewards naturally seem to belong to the solely responsible person producing a positive or negative outcome. In a group decision where responsibility is shared, however, the outcome may be shared among group members or given to one particular group member (such as a group leader, or the group’s representative, or to a particular person the collective decision has been made for). Responsibility underlies ownership: control and intent of an action, which are directly associated with responsibility attributions ^1, 2^, also predict whether a person is perceived as the owner of an object ^16^. Moreover, a person is attributed outcomes based on how much they ’deserve’ that outcome, an effect referred as the ’entitlement effect’ ^17^. If attributions of responsibility predict ownership, is the opposite true? Something that is owned does have a special value in the eyes of its owner. This has been particularly demonstrated with the ’endowment effect’ where participants value more positively and prefer to keep goods that are given to them ^18^. Ownership could, in addition to changing the value of the owned outcome, change the sense of responsibility for that outcome. This change in responsibility as a consequence of ownership would also be consistent with the ’Just World Hypothesis’ ^19^ in which people retroactively ascribe responsibility to people for the situations they are in.

**Figure 1.**
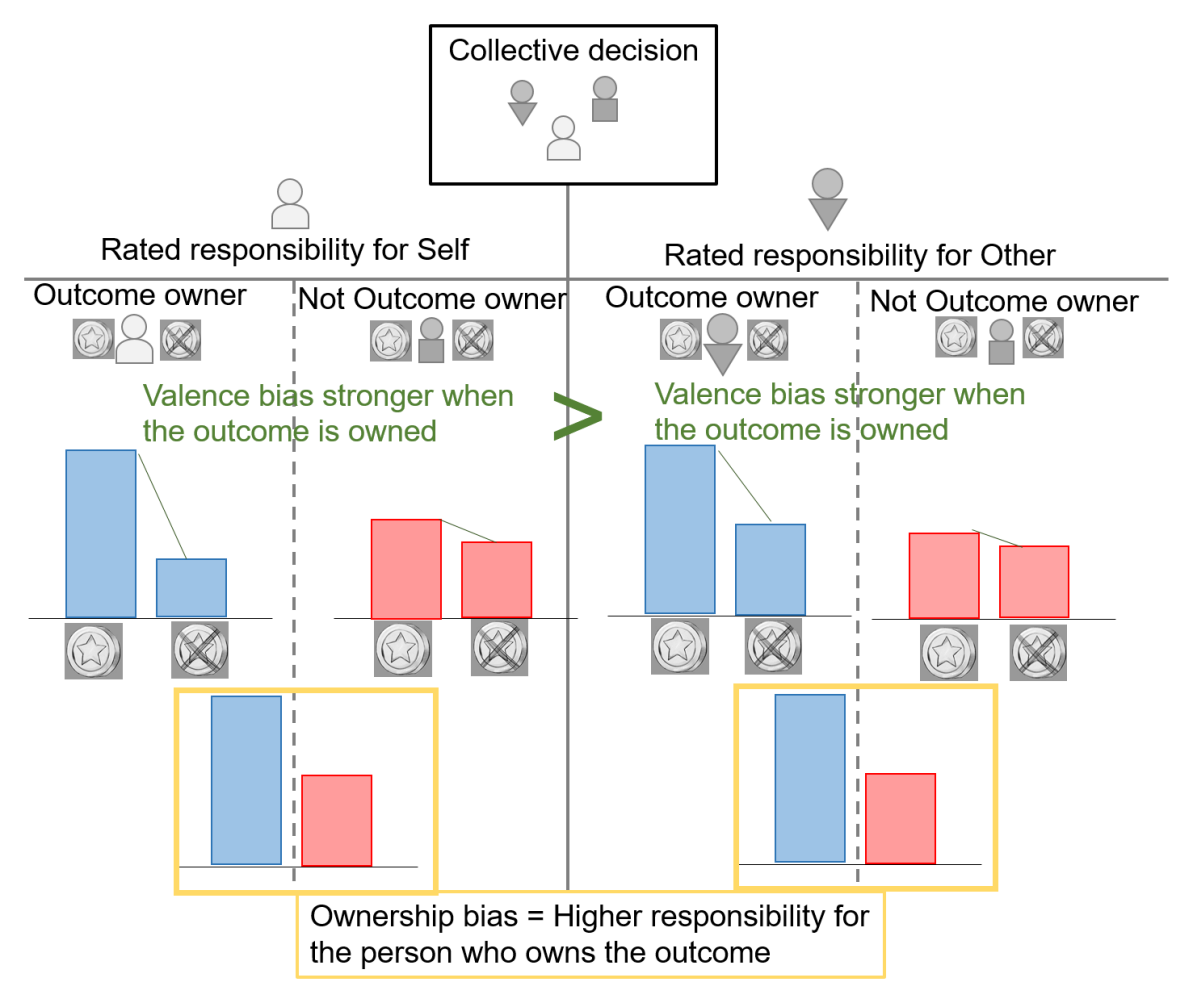
Expected responsibility ratings when people judge their own responsibility (self) and the responsibility of another group member (Other). The group member that receives (blue) vs does not receive (red) the outcome is rated as more responsible – in yellow: ownership bias, present both for responsibility ratings of Self and Other (Main effect of ownership). The valence bias (green) predicts higher responsibility for reward vs no reward (Main effect of outcome valence). This valence bias is predicted to be stronger when the rated person owns the outcome (OwnershipXOutcome valence interaction). This stronger valence bias for owned vs not owned outcome is also predicted to be more important for Self vs Other ratings (OwnershipXOutcome valenceXRated(self/other) interaction).

Here we would like to address 1) whether outcome ownership changes attributions of responsibility, and 2) whether the ‘valence bias’, i.e., increased responsibility for positive versus negative outcomes - that appears both when judging one’s own (self-serving bias) and another person’s (other-serving bias) responsibility - depends on the judged person being the owner of the outcome or not. We investigate this question in a group decision-making context, where only one member receives the outcome in each round. Participants will perform an online task where they make collective decisions through majority votes, then one member of the group receives the outcome: either a reward or no reward. Finally they rate the responsibility of all group members for the positive or negative outcome. This paradigm will allow us to address both questions stated above: 1) by investigating whether responsibility attribution increases when a group member receives the outcome versus does not receive the outcome, although the control over the outcome is exactly similar – referred to as the ‘ownership bias’. 2) By checking whether the valence bias of higher responsibility ratings for positive outcomes depends on whether the judged person receives the outcome of the group decision or not. In other words, in this second point, we aim to answer the question: do people exhibit the same valence bias when judging the responsibility for an outcome that is attributed not to them, but to another member of their group? Do they exhibit the same valence bias when judging the responsibility of another group member who received vs did not receive the outcome? (See Figure 1 for the predicted effects)

The results of the study will allow assessment of the link between outcome ownership and responsibility judgements, and comparison of how the valence bias for self and other is affected by outcome ownership. It may help identify biases in group responsibility attributions based on how the outcome is distributed.

The literature predominantly focuses on questions such as ’who deserves a specific positive or negative outcome based on contributions and actions?’. However, it is often the case that specific people, powerful leaders for example, receive outcomes for actions they were probably only partially or not responsible for. The work here investigates how observing a person getting an outcome can change responsibility attributions, possibly explaining how particular people may end up receiving all the credit for a collective work, both in their own eyes and others’.

## Methods

### Materials

Experiments were custom-written in HTML / CSS / JavaScript using the jsPsych framework ^20^ and undertaken by participants over the internet using their own devices. The experiment has to be performed on a desktop using a recent Google chrome or mozilla firefox browser. A demonstration version of the experiment is available at https://tinyurl.com/r-by-r-3/ATTRRESP/?demo=Y

### Procedure

The entry point to the study is through recruitment on the Prolific ( https://prolific.ac/) participant recruitment platform. The study is approved by the UCL Research Ethics Committee as Project ID Number: 5375/001. The only eligibility criterion is that participants have to be aged 18–40years. After accepting the study, they are forwarded to the experiment website. They first read an information page including ethics and data protection information. The next page is a consent form that is written in the form of sentences next to tick boxes, participants had to tick the four boxes in order to proceed: By checking the boxes below, I agree that:

1. I have carefully read the information page.
2. I have been given contact details of the researcher to ask any question or discuss the study.
3. I understand that I am free to withdraw at any time, without giving a reason, and without incurring any penalty.
4. I am over 18 years of age.

**Figure 2.**
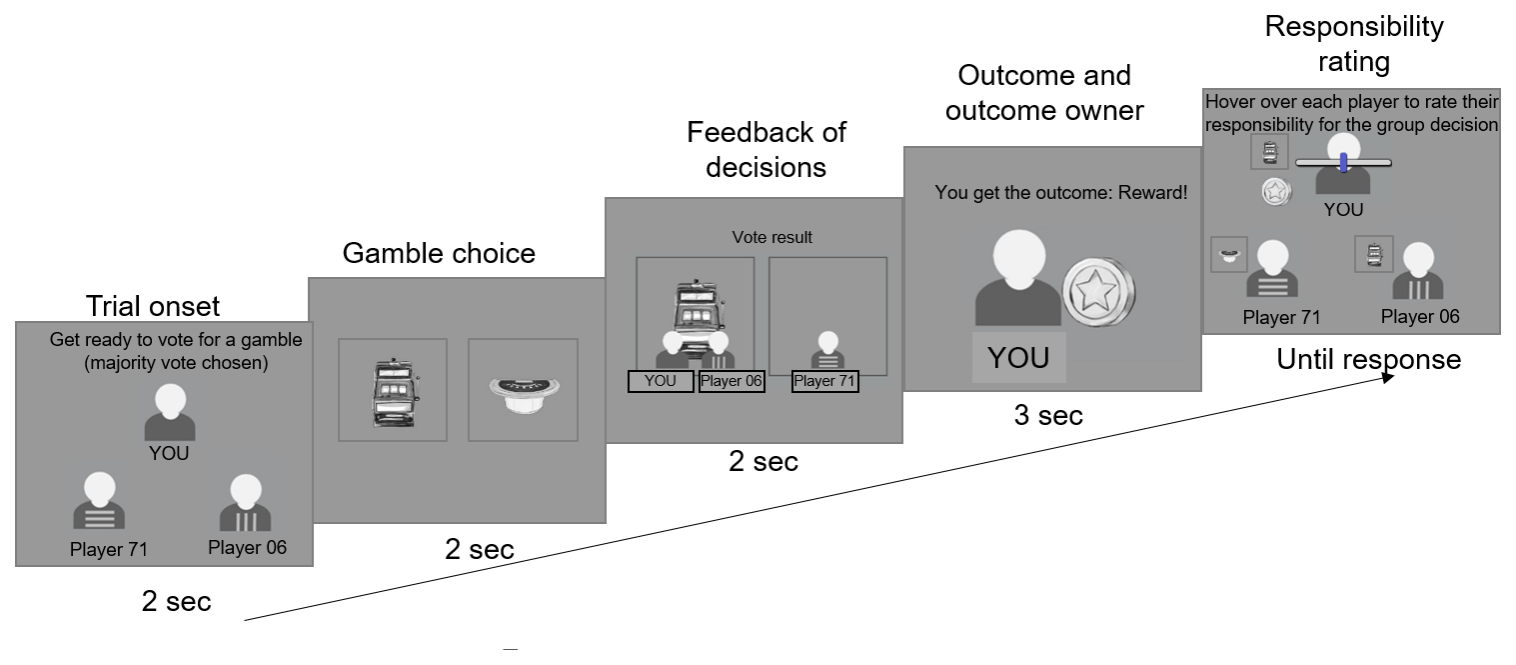
Experimental paradigm. At each trial, participants first see the group for 2 sec, then see the pair of gambles and have to pick one of the gambles by clicking on it within 2 sec. Afterwards, they see which gamble they and the other members of their group picked for 2 sec (Here the participant and Player 71 picked the left gamble that is therefore the majority choice, i.e. the group choice, while player 06 chose the right gamble). Following this, they see which player received the outcome, and whether that player was rewarded or not rewarded (Here, the participant receives the outcome and is rewarded). Finally, they have to rate the responsibility of each player for the outcome with no time limit except that once they finish rating the last player, the experiment continues to the next trial.

The experiment begins with detailed instruction pages which describe the structure of each round in the game, with screenshots of each stage, followed by two training trials. Once they have read the instructions and familiarised themselves with the game, participants begin the main experiment, which consists of 3 blocks of 24 trials. The 24 trials are a randomised sequence of 2 repetitions of each of the 12 unique trial types (as defined by whether the outcome is good/neutral; whether the participant is in the majority/minority; and which of the three players receives the outcome). The structure for each trial is shown in Figure 2. 

Each trial begins with a display of the three players lasting 2s. This is followed by a screen lasting 2s in which the participant selects one of two gamble images. Gamble images are selected from a collection of 20 hand-drawn images of gambling devices and paraphernalia, and each pairing of images in each trial is unique. Participants are told the different gambles have different probabilities of winning and losing and that they should try and pick the one that has the higher chances of reward. However, unbeknownst to the participants, which gamble is selected has no actual influence on the outcome of the trial, which is predetermined. Once the participant selects a gamble their player icon is drawn below that option.

If the participant has not selected a gamble by the end of the 2s choice window, the rest of the trial plays out invisibly behind a warning message which tells the participant that they have failed to make a choice in time. If the participant did select a gamble, the next screen is a 2s display of the votes from all three players indicating which gamble is to be selected. The gamble which received 0 or 1 votes is removed, and an 1.25s animation follows in which the gamble is allocated to one of the players and its outcome is shown (a coin for a rewarded trial or a coin with a cross through it for an unrewarded trial). The outcome then remains on the screen for 3s, showing both the valence (good/neutral) of the outcome and the player to whom it has been allocated.

Finally, all the players’ icons are restored to the screen (and next to each player is shown the gamble that they chose and their outcome if they had received the outcome on that trial). Participants assess each player’s responsibility in the order that they pick: if they drag the mouse toward one of the players, the slider for that player appears and they indicate the responsibility on this slider. The responsibility rating phase lasts until the participant submitted responsibility ratings for all the players.

Once all 72 experimental trials have been completed, participants are debriefed, thanked, and returned to Prolific. Payment (£3.5) follows once all participants have completed the study and bonuses have been calculated. The bonus is given based on one randomly selected trial: if that trial is a ’reward’ trial then they are allocated the bonus of £0.5.

### Pilot experiments

The design for the study was developed over the course of three pilot experiments. The final pilot experiment used the method of the main study. We wish to pursue two questions: 1) Does being the outcome recipient produce a difference in perceived responsibility? 2) Is the tendency to attribute more responsibility for good outcomes (valence bias) more pronounced for the outcome recipient’s perceived responsibility?

We will also explore, though in a more limited manner, whether the extent to which an agent’s responsibility for a group decision is affected by whether they were in the majority or minority.

Pilot experiments are seldom useful for giving answers to scientific questions ^21^, although the pilot experiments reported here may be sufficiently large (N = 47, 43, and 56) to give an indication.

Pilot experiments were preregistered where possible, and analyses and links to preregistration details, methods and results are available as follows: Pilot 1 - https://tinyurl.com/r-by-r-3/analysis/pilot-1.html; Pilot 2 - https://tinyurl.com/r-by-r-3/analysis/pilot-2.html; and Pilot 3 - https://tinyurl.com/r-by-r-3/analysis/pilot-3.html. The source code, data, and analysis results for the Pilot experiments can also be found via Zenodo ^22^.

Pilot Experiments 1 and 2 used groups of 5 players (of which the participant was one), had only one player’s responsibility rated on each trial, and did not show information about which players voted for which gamble. In other respects they were similar to Pilot Experiment 3, which used the design proposed for the main study and is detailed in the Procedure section.

We observed that pilot participants consistently provided higher responsibility ratings for good as compared to neutral outcomes, both for themselves (Pilots 1 and 3) and others (Pilots 2 and 3). We also observed that the outcome recipient was seen as having a greater responsibility for the joint decision than the other players (all Pilots). Finally, we saw that the increased attribution of responsibility for good over neutral outcomes was greater when the rated person was the recipient of the outcome (all Pilots).

### Bayesian statistics

We explored the use of Bayesian statistics in the pilot experiments, but the results of the analyses proved difficult to interpret. In the power analysis and planned analysis of future data we therefore use the frequentist ANOVA.

### Power analysis

We conducted a power analysis to determine how many participants we would need to detect effects of half the size found in the third pilot experiment. The effect for determining power was the most complicated interaction we were interested in, namely the three-way interaction which indicates a different size of effect for the valence bias increase when the outcome is owned and participants are judging the self vs another player. Power analysis was performed using custom simulation code written in R version 4.0.2 ^23^. Participants analysed in Pilot 3 were sampled randomly (with replacement) up to *N* participants. Each of the *N* participants were then used as the basis of a generative model by extracting parameters from linear regression on the participant’s pilot data. The effect size of the three-way interaction was replaced with a draw from a normal distribution with mean *E* and standard deviation equal to that observed in the pilot data (0.44). A grid search was conducted over some plausible values of *N* and *E* with 1000 simulations/cell. True (or false for *E* = 0) positive rates were calculated by running ANOVA on the data for each simulation. The power analysis indicated that 500 participants would provide around 93% power to detect an effect size of *d* = -0.07 (half of the effect size identified in Pilot 3, *d* = -0.14).

### Future analysis plan

First, data will be excluded based on the following rules:

- All data for subsequent attempts from participants attempting the experiment more than once will be dropped.
- All data for participants failing to choose a gamble on 10 or more trials will be dropped.
- All data for participants who fail to use both response buttons in choosing gambles in each block will be dropped.
- All data for participants whose data does not include all 72 trials will be dropped.
- Trials where the participant failed to respond will be dropped.
- Trials where participants stayed longer than 15 sec on the responsibility rating screen will be dropped.
- All data for participants who stayed more than 15sec on the responsibility rating screen on 10 or more trials will be dropped.

The data will then be converted into a long format where each trial provides three observations - one responsibility rating for each player. The data will be analysed (without further truncation or cleaning) using a 2 (reward vs no reward outcome) × 2 (rated player did vs did not get outcome) × 2 (rated player is vs is not participant) ANOVA with the alpha level set at .05. Participant-level data will be collapsed into means for each of the six contingencies for the purposes of ANOVA.

The draft of the analysis script, as it stood at Stage 1 submission time, is available at https://github.com/mjaquiery/responsibility-by-reward/blob/2b0eb49760332f3121a9472dfd1cd2311e2121cd/analysis/main.Rmd.

## Study status

Three pilots experiments were conducted for this study. The main study and current pre-registered report went through a first round of peer-review. This is our revised version and we will wait for the study to be approved before conducting the study.

## Dissemination

The study results will be disseminated in internal lab meetings, national and international conferences.

## Data availability

### Underlying data

Data for the pilot experiments, along with data dictionaries describing the variables, are available in .csv format from ./data directory of the GitHub repository ( https://github.com/mjaquiery/responsibility-by-reward/tree/master/data) ^22^. 

Data are available under the terms of the Creative Commons Attribution 4.0 International license (CC-BY 4.0).

## Software availability

Code for experiments available here: https://github.com/mjaquiery/responsibility-by-reward/tree/master/ATTRRESP

Archived code as at time of publication: http://doi.org/10.5281/zenodo.4452004 ^22^ License: CC-BY 4.0
