# Supplementary material for: Stage 1 Registered Report: How responsibility attributions to self and others relate to outcome ownership in group decisions
Source: Wellcome Open Res. 2021 May 5;6:24. Originally published 2021 Feb 5. [Version 2] doi: 10.12688/wellcomeopenres.16480.2 (PMC8258704; doi:10.12688/wellcomeopenres.16480.2)
Supplement: Peer review history

# Peer review: Reviewer response for version 2

**DOI:** 10.21956/wellcomeopenres.18607.r43680

**Published:** 2021-07-05

**Authors:** John Dewey

**Affiliations:** 1 Department of Psychological Sciences, University of North Georgia, Dahlonega, GA, USA

The amendments look good.

Is the study design appropriate for the research question?

Yes

Have the authors pre-specified sufficient outcome-neutral tests for ensuring that the results obtained can test the stated hypotheses, including positive controls and quality checks?

Yes

Is the rationale for, and objectives of, the study clearly described?

Yes

Are sufficient details of the methods provided to allow replication by others?

Yes

Are the datasets clearly presented in a useable and accessible format?

Yes

Reviewer Expertise:

Sense of agency and responsibility attributions

I confirm that I have read this submission and believe that I have an appropriate level of expertise to confirm that it is of an acceptable scientific standard.

# Peer review: Reviewer response for version 2

**DOI:** 10.21956/wellcomeopenres.18607.r43679

**Published:** 2021-06-30

**Authors:** Nura Sidarus

**Affiliations:** 1 Royal Holloway University of London, Egham, UK

The authors' detailed response is appreciated and the revisions have addressed the key concerns, with the manuscript being improved.

 The addition of figure 1, outlining the research design and predictions, is also appreciated, however, I believe its current form is considerably confusing, in terms of what’s depicted and how, the definitions of concepts, how the various predictions are depicted, etc.

 For some examples: the equal green text on both sides of the middle line is confusing when intending to depict an interaction; it seems the authors predict a main effect of ownership (fig legend), but the repetition of equal bars on both sides rather imply a prediction of *no* interaction between the ownership bias and who is being rated; the 3-way interaction prediction isn’t very evident from the figure (e.g. the blue bar for the outcome owner with positive outcomes seems the same height on the self vs other rated side, whereas a plausible main effect of who’s rated would predict lower ratings for other). Perhaps starting from the main effect of ownership, and working downwards towards the 3-way interaction, or depicting the various effects separately, could facilitate interpretation, or the authors may find other solutions.

 I understand this is a difficult figure to draw to ensure clarity, but I trust that it can be revised and it will be a helpful addition to the paper.

Is the study design appropriate for the research question?

Yes

Have the authors pre-specified sufficient outcome-neutral tests for ensuring that the results obtained can test the stated hypotheses, including positive controls and quality checks?

Yes

Is the rationale for, and objectives of, the study clearly described?

Partly

Are sufficient details of the methods provided to allow replication by others?

Yes

Are the datasets clearly presented in a useable and accessible format?

Yes

Reviewer Expertise:

Cognitive neuroscience, sense of agency (/responsibility attribution), metacognition, decision making

I confirm that I have read this submission and believe that I have an appropriate level of expertise to confirm that it is of an acceptable scientific standard.

# Peer review: Reviewer response for version 1

**DOI:** 10.21956/wellcomeopenres.18145.r42948

**Published:** 2021-04-06

**Authors:** Nura Sidarus

**Affiliations:** 1 Royal Holloway University of London, Egham, UK

This article investigates how responsibility judgements are influenced by whether action outcomes are desirable or not, and how that effect is influenced by being the owner, or recipient, of the outcomes, as well as whether one is judging oneself or others. Pilot studies support the presence of an ownership bias, where responsibility is higher for the outcome owner than non-owners, and for a valence bias for the outcome owner, which is seen as more responsible for positive than negative outcomes, which is larger for oneself (self-serving) than for others (other-serving bias). These are very interesting research questions and methods, bridging between fields of research that refer to related concepts, combining information to arrive at new hypotheses and providing a strong way to operationalise and test the ideas. The transparent sharing of the details of data collection and analysis already performed is commendable, helping to clarify the research ideas and demonstrate the researchers’ strengths to competently conduct the planned research. The pilot studies provide a strong grounding for the planned study and for the detailed power analysis based on simulations to estimate the necessary sample size for the main study to test the relevant 3-way interaction. I believe the study should be completed and the manuscript published, but that the authors should first consider the following points, which essentially concern revisions to the manuscript.

  

1. The various ways of rephrasing the two research questions, especially for Q2, can seem to imply different things. Some of the writing seems to imply that the biases will exclusively appear for the owner, whereas other parts imply rather that the extent of the bias will be moderated by ownership. Some of the questions phased seem a bit different from the initial point, and somewhat confusing regarding what comparisons would test them. For example:
*“* *whether outcome ownership is **necessary** for the self-serving and/or other-serving bias (more responsibility for positive versus negative outcomes) to appear.* *“*
  [the bias] *“* *depends on who gets the outcome* *” *
  “ *In other words, in this second point, we aim to answer the question:*
*1) do people exhibit the same self-serving bias when judging the responsibility for an outcome that is attributed to another member of their group?*
*2) Do they also still exhibit an ‘other-serving’ bias when judging the responsibility of another group member who did not receive the outcome?*“ (p.3)
  Phrasing the question as whether outcome ownership is “necessary” for a self/other serving bias leads me to wonder how that could really be addressed here if there’s always one person receiving the outcome. If the other-serving bias is similar to, even if smaller, than the self-serving one, then if a person receives the outcome, one would still predict some valence bias to be present. Therefore, I’d think the “necessity” of outcome ownership for the bias could only be demonstrated by including a condition in which “no one benefits”, as a hypothesised “control” condition in which no valence bias would be present.
  Instead, the authors may rather aim to test whether there’s an interaction between the effect of valence and being the owner vs not – as stated in the abstract - in more specific terms relating to the current operationalisation.
  Some of the confusion seems related to whether the “self/other -serving” bias is assumed to appear as a function of who’s being judged vs. who gets the outcome. Sometimes it seems “self-serving bias” being used to refer to when judging the self as having greater responsibility for positive over negative outcomes only when it’s the “owner” of outcomes, vs. asking whether that happens regardless of ownership. Perhaps using more general terms like “valence bias”, and perhaps “self bias” (to indicate a bias to judging higher responsibility for the self than other regardless of outcome valence), could make it easier to distinguish when that’s expected to be affected by who’s being judged, vs. who gets the outcome.
  What is intended is made more evident from their pilot analysis linked, but the introduction can be improved to ensure the key questions and hypotheses are clearly stated and it is evident how the data could support or not different hypotheses (i.e. which comparisons test the hypotheses). It may also be useful to draw graphs of the hypothesised results depicting the patterns of results/interactions that would support different, or competing, hypotheses. This is important for demonstrating the adequacy of the study design and of the conclusions that can be drawn from the results.
2. The authors stated that the primary analysis is a 2x2x2 ANOVA, but their detailed analysis scripts don’t start with that test, but rather by addressing a series of more specific questions with more specific tests (e.g. t-test on being the owner vs not, which would seem equivalent to the main effect in the ANOVA). While I understand their logic, seems more consistent to start with the planned primary statistical test, describe that and then run whichever other tests, or rewrite the planned analysis in the manuscript. Relatedly, it seems the authors plan to run Bayesian statistical tests, but only note in the manuscript using frequentist tests, but I don’t see why they wouldn’t say in advance they plan to use both.
3. Methods, state: “Finally, all the players’ icons are restored to the screen and each player’s responsibility is assessed by the participant by clicking on an icon and then indicating the responsibility using a slider. The responsibility rating phase lasts until responsibility ratings have been submitted for each player.“ – please add clarification of whether the order of ratings is fixed (e.g. always “You”, then “Player 06”, then “Player 71”) or randomised.
4. Text on the task figure is too small to read even when enlarged on the website.

Is the study design appropriate for the research question?

Yes

Have the authors pre-specified sufficient outcome-neutral tests for ensuring that the results obtained can test the stated hypotheses, including positive controls and quality checks?

Yes

Is the rationale for, and objectives of, the study clearly described?

Partly

Are sufficient details of the methods provided to allow replication by others?

Yes

Are the datasets clearly presented in a useable and accessible format?

Yes

Reviewer Expertise:

Cognitive neuroscience, sense of agency (/responsibility attribution), metacognition, decision making

I confirm that I have read this submission and believe that I have an appropriate level of expertise to confirm that it is of an acceptable scientific standard, however I have significant reservations, as outlined above.

# Response

**Published:** 2021-04-23

**Authors:** Marwa El Zein

**Affiliations:** University College London, UK

*This article investigates how responsibility judgements are influenced by whether action outcomes are desirable or not, and how that effect is influenced by being the owner, or recipient, of the outcomes, as well as whether one is judging oneself or others. Pilot studies support the presence of an ownership bias, where responsibility is higher for the outcome owner than non-owners, and for a valence bias for the outcome owner, which is seen as more responsible for positive than negative outcomes, which is larger for oneself (self-serving) than for others (other-serving bias). These are very interesting research questions and methods, bridging between fields of research that refer to related concepts, combining information to arrive at new hypotheses and providing a strong way to operationalise and test the ideas. The transparent sharing of the details of data collection and analysis already performed is commendable, helping to clarify the research ideas and demonstrate the researchers’ strengths to competently conduct the planned research. The pilot studies provide a strong grounding for the planned study and for the detailed power analysis based on simulations to estimate the necessary sample size for the main study to test the relevant 3-way interaction. I believe the study should be completed and the manuscript published, but that the authors should first consider the following points, which essentially concern revisions to the manuscript.*

 We thank the reviewer for acknowledging the interest of the research question and methods and for providing important revisions that improve the paper.

**

*The various ways of rephrasing the two research questions, especially for Q2, can seem to imply different things. Some of the writing seems to imply that the biases will exclusively appear for the owner, whereas other parts imply rather that the extent of the bias will be moderated by ownership. Some of the questions phased seem a bit different from the initial point, and somewhat confusing regarding what comparisons would test them. For example:*

*“whether outcome ownership is necessary for the self-serving and/or other-serving bias (more responsibility for positive versus negative outcomes) to appear. “*

*[the bias] “depends on who gets the outcome”*

*“ In other words, in this second point, we aim to answer the question:*

*1) do people exhibit the same self-serving bias when judging the responsibility for an outcome that is attributed to another member of their group?*

*2) Do they also still exhibit an ‘other-serving’ bias when judging the responsibility of another group member who did not receive the outcome?“ (p.3)*

*Phrasing the question as whether outcome ownership is “necessary” for a self/other serving bias leads me to wonder how that could really be addressed here if there’s always one person receiving the outcome. If the other-serving bias is similar to, even if smaller, than the self-serving one, then if a person receives the outcome, one would still predict some valence bias to be present. Therefore, I’d think the “necessity” of outcome ownership for the bias could only be demonstrated by including a condition in which “no one benefits”, as a hypothesised “control” condition in which no valence bias would be present.*

*Instead, the authors may rather aim to test whether there’s an interaction between the effect of valence and being the owner vs not – as stated in the abstract - in more specific terms relating to the current operationalisation.*

*Some of the confusion seems related to whether the “self/other -serving” bias is assumed to appear as a function of who’s being judged vs. who gets the outcome. Sometimes it seems “self-serving bias” being used to refer to when judging the self as having greater responsibility for positive over negative outcomes only when it’s the “owner” of outcomes, vs. asking whether that happens regardless of ownership. Perhaps using more general terms like “valence bias”, and perhaps “self bias” (to indicate a bias to judging higher responsibility for the self than other regardless of outcome valence), could make it easier to distinguish when that’s expected to be affected by who’s being judged, vs. who gets the outcome.*

*What is intended is made more evident from their pilot analysis linked, but the introduction can be improved to ensure the key questions and hypotheses are clearly stated and it is evident how the data could support or not different hypotheses (i.e. which comparisons test the hypotheses). It may also be useful to draw graphs of the hypothesised results depicting the patterns of results/interactions that would support different, or competing, hypotheses. This is important for demonstrating the adequacy of the study design and of the conclusions that can be drawn from the results.*

 We thank the reviewer for noting this important point about the vocabulary we use, as we now realize how our formulation of hypotheses may have been unclear. We rephrase our sentences to avoid using ‘necessary’, because indeed, we aim to test the interaction between the valence effect and being the outcome owner or not. We adopt the terminology suggested ‘valence bias’ and ‘ownership bias’ and thank the reviewer for these suggestions as we agree they are more accurate to clarify the expected effects. All the cited sentences are re-phrased as follows and we hope our edits to the introduction clarified our questions:

*‘whether the ‘valence bias’, i.e., increased responsibility for positive versus negative outcomes, - that appears both when judging one’s own (self-serving bias) and another person’s (other-serving bias) responsibility - depends on the judged person being the owner of the outcome or not.’*

*‘…depends on whether the judged person receives the outcome of the group decision or not.’*

*‘In other words, in this second point, we aim to answer the question: do people exhibit the same valence bias when judging the responsibility for an outcome that is attributed not to them, but to another member of their group? Do they exhibit the same valence bias when judging the responsibility of another group member who received vs did not receive the outcome?’*

 We also followed the reviewer’s advice to add a figure with the hypothesized results that can clarify our hypotheses and expected results (Figure 1).

*The authors stated that the primary analysis is a 2x2x2 ANOVA, but their detailed analysis scripts don’t start with that test, but rather by addressing a series of more specific questions with more specific tests (e.g. t-test on being the owner vs not, which would seem equivalent to the main effect in the ANOVA). While I understand their logic, seems more consistent to start with the planned primary statistical test, describe that and then run whichever other tests, or rewrite the planned analysis in the manuscript. Relatedly, it seems the authors plan to run Bayesian statistical tests, but only note in the manuscript using frequentist tests, but I don’t see why they wouldn’t say in advance they plan to use both.*

 We follow the reviewer’s advice to start with the ANOVA and then unfold the rest of our results. This should now be visible in all the pilots’ analysis scripts as well as the main analysis script.

 Concerning Bayesian statistics, we add the following statement in the text to explain why we do not plan to use them:

*‘Bayesian statistics*

*We explored the use of Bayesian statistics in the pilot experiments, but the results of the analyses proved difficult to interpret. In the power analysis and planned analysis of future data we therefore use the frequentist ANOVA.’*

*Methods, state: “Finally, all the players’ icons are restored to the screen and each player’s responsibility is assessed by the participant by clicking on an icon and then indicating the responsibility using a slider. The responsibility rating phase lasts until responsibility ratings have been submitted for each player.“ – please add clarification of whether the order of ratings is fixed (e.g. always “You”, then “Player 06”, then “Player 71”) or randomised.*

 We hope we have clarified this by changing the sentence to *“Finally, all the players’ icons are restored to the screen (and next to each player is shown the gamble that they chose and their outcome if they had received the outcome on that trial). Participants assess each player’s responsibility in the order that they pick: if they drag the mouse toward one of the players, the slider for that player appears and they indicate the responsibility on this slider. The responsibility rating phase lasts until the participant submitted responsibility ratings for all the players.’’*

**

*Text on the task figure is too small to read even when enlarged on the website.*

 We apologize for this and have fixed the figure so that the text can be easily read.

# Peer review: Reviewer response for version 1

**DOI:** 10.21956/wellcomeopenres.18145.r42502

**Published:** 2021-02-16

**Authors:** John Dewey

**Affiliations:** 1 Department of Psychological Sciences, University of North Georgia, Dahlonega, GA, USA

The authors propose to investigate how outcome ownership influences attributions of responsibility for group decisions (i.e. if you personally receive a payment based on the decisions of the group, do you feel more responsible for that group decision?). The idea behind the proposal is interesting. I do have a few minor concerns regarding the details of the methodology, however:

- The instructions in the demo version say “Your aim is to find which gambles are likely to make you win and choose them. Please be aware that there is no link beween the type of image depicted (roulettes, dice, cards) and the actual probablity to win.”
  There are a couple of typos in the instructions. More importantly though, if I was a participant in this study, I would wonder how I’m supposed to find which gambles are likely to make me win if there’s no link between the images and the probability of winning. It seems like you’re admitting to participants upfront that they will just be guessing at random. Maybe you should omit that first sentence and let participants wonder if there is an optimal strategy to discover, similar to the Iowa Gambling task. I think this would add more variance to your data, which might seem like a bad thing, but it would also reduce the chances of people mindlessly applying a response set. Specifically, if participants know that the image doesn’t matter, they decide on a simple rule like “the people in the majority are always responsible”, or “we’re all equally responsible for every outcome, since it’s all just random anyway”.
- Because the experiment has a quick pace, I think participants will often fail to notice (or quickly forget) who voted for which outcome. When you present the screen for judging the responsibility of each actor, it might be good to add a visual reminder of how each person voted.
- Under the ‘Data Availability’ section, the GitHub link leads to the /ATTRRESP subdirectory. I believe it should lead instead to the /data subdirectory.

Is the study design appropriate for the research question?

Yes

Have the authors pre-specified sufficient outcome-neutral tests for ensuring that the results obtained can test the stated hypotheses, including positive controls and quality checks?

Yes

Is the rationale for, and objectives of, the study clearly described?

Yes

Are sufficient details of the methods provided to allow replication by others?

Yes

Are the datasets clearly presented in a useable and accessible format?

Yes

Reviewer Expertise:

Sense of agency and responsibility attributions

I confirm that I have read this submission and believe that I have an appropriate level of expertise to confirm that it is of an acceptable scientific standard, however I have significant reservations, as outlined above.

# Response

**Published:** 2021-04-23

**Authors:** Marwa El Zein

**Affiliations:** University College London, UK

*The authors propose to investigate how outcome ownership influences attributions of responsibility for group decisions (i.e. if you personally receive a payment based on the decisions of the group, do you feel more responsible for that group decision?). The idea behind the proposal is interesting.*

 We thank the reviewer for noting the interest of our work.

*I do have a few minor concerns regarding the details of the methodology, however:*

*The instructions in the demo version say “Your aim is to find which gambles are likely to make you win and choose them. Please be aware that there is no link beween the type of image depicted (roulettes, dice, cards) and the actual probablity to win.”*

*There are a couple of typos in the instructions. More importantly though, if I was a participant in this study, I would wonder how I’m supposed to find which gambles are likely to make me win if there’s no link between the images and the probability of winning. It seems like you’re admitting to participants upfront that they will just be guessing at random. Maybe you should omit that first sentence and let participants wonder if there is an optimal strategy to discover, similar to the Iowa Gambling task. I think this would add more variance to your data, which might seem like a bad thing, but it would also reduce the chances of people mindlessly applying a response set. Specifically, if participants know that the image doesn’t matter, they decide on a simple rule like “the people in the majority are always responsible”, or “we’re all equally responsible for every outcome, since it’s all just random anyway”.*

 We are sorry for the typos and hope we have corrected all of them. We completely agree with the point raised by the reviewer and we thank him for noticing that this instructions sentence can be understood in this way as we did not mean for it. What we meant to convey with ‘ *Please be aware that there is no link between the type of image depicted (roulettes, dice, cards) and the actual probability to win.’* was related to the ‘type’ of the image not the image itself: if a participant is familiar with gambling and knows that in real life, roulettes have higher chances to win than dice for example, this doesn’t apply in the experiment. Rather, different images of gambles are associated with different probabilities of winning independently of their chances of success in real life. To avoid this misunderstanding, we followed the reviewer’s advice and removed this sentence keeping ‘You *will have to decide which gamble to choose by pressing on the left or right gamble. The images will only appear for 2 seconds, and you only have 2 seconds to make your choice. Each gamble has different probabilities of winning and losing. Your aim is to find which gambles are likely to make you win and choose them.’*

**

*Because the experiment has a quick pace, I think participants will often fail to notice (or quickly forget) who voted for which outcome. When you present the screen for judging the responsibility of each actor, it might be good to add a visual reminder of how each person voted.*

 We followed the reviewer’s recommendation to add a reminder at the time of the ratings of who voted for which gamble next to each group member, as well as the obtained outcome next to the person who received it. We changed Figure 1 accordingly and this is now also visible in our demo experiment.

**

*Under the ‘Data Availability’ section, the GitHub link leads to the /ATTRRESP subdirectory. I believe it should lead instead to the /data subdirectory.*

 Thank you for noting this, we have fixed it.
